# Assessment of predictors of response and long-term survival of patients with neuroendocrine tumour treated with peptide receptor chemoradionuclide therapy (PRCRT)

**DOI:** 10.1007/s00259-014-2788-5

**Published:** 2014-05-21

**Authors:** G. Kong, M. Thompson, M. Collins, A. Herschtal, M. S. Hofman, V. Johnston, P. Eu, M. Michael, Rodney J. Hicks

**Affiliations:** 1 Centre for Cancer Imaging, Peter MacCallum Cancer Centre, St Andrew’s Place, East Melbourne, VIC 3002 Australia; 2 Department of Biostatistics and Clinical Trials, Peter MacCallum Cancer Centre, Melbourne, VIC Australia; 3 Department of Medicine, The University of Melbourne, Melbourne, VIC Australia; 4 Division of Cancer Medicine, Neuroendocrine Tumour Unit, Peter MacCallum Cancer Centre, Melbourne, VIC Australia

**Keywords:** ^177^Lu-Octreotate, Neuroendocrine, 5-FU chemotherapy, Response, Predictive factors, Survival

## Abstract

**Purpose:**

To review the response and outcomes of ^177^Lu-DOTA-octreotate chemoradionuclide therapy (LuTate PRCRT) in patients with neuroendocrine tumour (NET) expressing high levels of somatostatin receptors with uncontrolled symptoms or disease progression.

**Methods:**

A total of 68 patients (39 men; 17 – 76 years of age) who had completed an induction course of at least three cycles of LuTate PRCRT between January 2006 and June 2010 were reviewed. Ten patients were treated for uncontrolled symptoms and 58 had disease progression despite conventional treatment. The majority had four induction LuTate cycles (median treatment duration 5 months and cumulative activity 31 GBq), and 63 patients had concomitant 5-FU radiosensitizing infusional chemotherapy. Factors predicting overall survival were assessed using the log-rank test and Cox proportional hazards regression.

**Results:**

Of those treated for uncontrolled symptoms, 70 % received benefit maintained for at least 6 months after treatment. Among patients with progressive disease 68 % showed stabilization or regression on CT, 67 % on molecular imaging and 56 % biochemically up to 12 months after treatment; 32 patients died. Overall survival rates at 2 and 5 year were 72.1 % and 52.1 %, respectively. Median overall survival was not estimable at a median follow-up of 60 months (range 5 – 86 months). Nonpancreatic primary sites, dominant liver metastases, lesion size <5 cm and the use of 5-FU chemotherapy were statistically significantly associated with objective response. A disseminated pattern and a high disease burden (whole-body retention index) were associated with an increased risk of death. Objective biochemical, molecular imaging and CT responses were all associated with longer overall survival.

**Conclusion:**

A high proportion of patients with progressive NET or uncontrolled symptoms received therapeutic benefit from LuTate with concomitant 5-FU chemotherapy. The achievement of objective biochemical, molecular or CT responses within 12 months was associated with improved overall survival. Patients with a primary pancreatic site and larger lesions (>5 cm) appeared to have lower objective response rates and may need a more aggressive treatment approach.

**Electronic supplementary material:**

The online version of this article (doi:10.1007/s00259-014-2788-5) contains supplementary material, which is available to authorized users.

## Introduction

Neuroendocrine tumours (NET) comprise a heterogeneous group of neoplasms arising within the diffuse endocrine system with variable clinical behaviour depending on the grade of tumour. Well-differentiated NETs typically have a low proliferative index, a slow pattern of growth but can metastasize widely, and secrete hormones resulting in debilitating clinical symptoms. Poorly-differentiated disease with a high proliferative index can result in more rapidly progressive disease and early mortality. Most well-differentiated to moderately-differentiated tumours retain high expression of somatostatin receptors (SSTR), a characteristic that can be identified by molecular imaging and targeted therapeutically by peptide receptor radionuclide therapy (PRRT). Current guidelines support the use of PRRT in patients with unresectable grade 1 or 2 NET [1, 2] and high SSTR expression at known tumour sites.

 A number of radionuclides have been conjugated to various somatostatin analogues for PRRT. ^90^Y emits the most energetic beta particles and is associated with favourable responses but moderate toxicity, particularly including renal impairment [3, 4]. ^111^In emits the least energetic particles but, although associated with minimal significant side effects, is associated with low response rates [5, 6]. More recently, ^177^Lu-labelled PRRT has been used in a number of international centres, and has shown favourable responses with limited toxicity. Its relatively lower beta particle emission energy (when compared with ^90^Y) limits radiation exposure and toxicity to adjacent normal tissue, whilst still providing the benefit of the cross-fire effect [5, 7, 8].

 Based on the experience in Rotterdam [9], our institution started using ^177^Lu-DOTA-octreotate (LuTate) therapy in late 2005 for the treatment of patients with unresectable, symptomatic or progressive NET, not controlled with conventional therapy. In addition, our PRRT protocol also included the use of concomitant infusional 5-fluorouracil (5-FU) chemotherapy as a radiosensitizing agent to potentially further enhance therapeutic efficacy (peptide receptor chemoradionuclide therapy, PRCRT). This is based on our previous experience that combining 5-FU and high-activity ^111^In-octreotide therapy is safe, with enhanced efficacy particularly with symptomatic benefit in patients with NET [6]. We have also previously reported that LuTate is well tolerated by patients who have previously received ^111^In-pentreotide therapy, and can be safely combined with 5-FU chemotherapy without significant early or late bone marrow or renal toxicities [10]. We review here the therapeutic response to PRCRT with LuTate treatment with medium term to long-term follow-up, and assess any predictive factors associated with response and overall survival (OS) in patients with uncontrolled NET.

## Patients and methods

### Patients

A total of 68 patients (29 women, 39 men; median age 56 years, range 17 – 76 years) with NET who started and completed an induction course of at least three cycles of LuTate therapy between January 2006 and June 2010 were retrospectively reviewed. These patients were followed up to death or the study close date of 1 June 2013. Median follow-up, calculated using the reverse censoring method, was 60 months (5 – 86 months).

Eligibility criteria for LuTate therapy included high SSTR-expressing disease as reflected by positive SSTR scan (^111^In-octreotide SPECT/CT or ^68^Ga-octreotate PET/CT scan) with an uptake intensity greater than that of normal hepatic parenchyma, either uncontrolled symptomatic disease despite maximal conventional treatment or progressive disease within the previous 12 months as reflected by biochemical (chromogranin A level, CgA level), SSTR or CT imaging criteria. Patients with a Ki-67 index >10 % or with evidence of metabolically active disease on the FDG PET/CT scan were generally offered chemotherapy as first-line therapy, since these features are associated with higher grade, less well-differentiated disease and a poorer prognosis [11].

Exclusion criteria for PRRT included spatially discordant FDG-avid disease showing low or absent SSTR analogue avidity, decompensated carcinoid heart disease, glomerular filtration rate <30 ml/min, hypoalbuminaemia (<20 g/L), platelet count <50 × 10^9^/L or pancytopaenia (unless considered to be reflective of a paraneoplastic process), ECOG performance score 4, expected survival <3 months, and pregnancy.

All patients were treated on compassionate grounds under a Special Access Scheme (SAS) which allows treatment of patients with life-threatening diseases with experimental therapies that have demonstrated efficacy in international trials, provided that no other proven treatment is available. The use of SAS provisions was approved by our Institutional Ethics Committee and all patients provided written informed consent to undergo treatment and follow-up.

### Treatment regimen

^177^Lu was produced and transported to our institution from Europe (IDB Holland) as a radiochemical. The peptide octreotate (Erasmus Medical Centre, Rotterdam, Holland) was labelled with ^177^Lu by chelation to a DOTA molecule to form ^177^Lu-DOTA-octreotate (LuTate).

Each cycle of LuTate was administered intravenously on an outpatient day-case basis with premedication including granisetron and dexamethasone, and with renoprotective amino acid infusion (25 g lysine and 25 g arginine in 1 L normal saline) commencing 30 min before PRRT and continuing for 3 h thereafter. At 24 h after each treatment, patients underwent a whole-body posttherapy scan. A whole-body retention index based on the percentage of administered dose retained as shown on the post-LuTate whole-body images after the first cycle of treatment was calculated by acquiring anterior and posterior whole-body images using medium energy collimation. A reference sample of <5 MBq ^177^Lu was placed at the feet of the patient. Regions of interest were drawn around the body, the reference and a background area outside the patient, and the geometric mean of the background-corrected whole-body regions was calculated. The percentage retention was calculated as the ratio of whole-body counts to the reference counts multiplied by the ratio of the reference activity to the decay-corrected administered activity. While this generally reflects disease burden, it is recognized that impaired renal function may also influence retention.

The induction treatment regimen typically included four cycles of LuTate PRCRT 6 – 10 weeks apart. However, some patients with extensive bone disease received an initial cycle of ^111^In-octreotate followed by three LuTate cycles. We considered that the shorter particle path length of ^111^In compared to ^177^Lu may have provided better targeting of small bony lesions whilst limiting potential toxicity to the surrounding marrow. The second to fourth cycles were usually given with infusional 5-FU chemotherapy (200 mg/m^2^/24 h) through a peripherally inserted central catheter as a radiosensitizing agent, starting approximately 4 days before the day of PRRT administration, and continued for 3 weeks in total. Administration was stopped in the event of hand–foot syndrome or other acute toxicity. The number of cycles was increased in some patients with a large disease burden and decreased in patients with a relatively small volume of disease or comorbidity. Patients who demonstrated disease stabilization or improvement were offered ongoing maintenance therapy (usually a single LuTate cycle) every 6 – 18 months, guided by clinical, imaging and biochemical parameters pertinent to their initial presentation, response and comorbidities. Maintenance treatments were offered to patients with symptomatic or persisting disease with high SSTR expression (Krenning score 3 or 4, i.e. greater than hepatic uptake or at least equal to splenic uptake, respectively) who had shown a previous symptomatic or objective response (stability or improvement) to induction LuTate cycles. This was given “prophylactically” every 12 months, or earlier in the event of biochemical or imaging disease recrudescence.

### Follow-up

Patients received follow-up assessments at our institution or locally (especially for interstate patients) at 3 – 6 months, 6 – 12 months and >12 months after the last induction cycle. Evaluation included assessment of symptoms, biochemical markers, molecular imaging (repeating whichever SSTR imaging investigation had been performed as the baseline staging investigation) and CT. Symptomatic benefit was defined as an improvement in tumour-related symptoms based on the patient’s subjective report relative to baseline symptoms. A clinician conducted patient interviews at each follow-up with direct questioning as to whether tumour-related symptoms had ‘disappeared’ or ‘improved’, or were ‘stable’ or ‘worse’ compared to baseline. Standard international quality-of-life questionnaires were not routinely used in this cohort, but have since been implemented.

 Biochemical response was documented as the percentage change using CgA level just prior to the first LuTate cycle as baseline. Molecular imaging response was defined as stable, partial response (reduction in intensity or extent or the disappearance of abnormal uptake at some sites of disease), complete response (total disappearance of abnormal uptake by previous avid lesions) or progressive disease (increase in intensity or extent of previous abnormal uptake, or development of new avid lesions). ^111^In-Octreotate SPECT or ^68^Ga-octreotate PET images were assessed as indicating a response if they showed a decrease in uptake relative to hepatic and splenic activity representing a reduction in uptake by one or more points on the Krenning scale. Metabolic responses were assessed on the FDG PET images according to the Hicks criteria as detailed in the PERCIST paper by Wahl et al. [12, 13]. CT response was defined as stable, partial or complete response, or progression defined by Response Evaluation Criteria in Solid Tumors (RECIST 1.1). Minor response was also used to describe smaller size changes not meeting the criteria for partial response according to RECIST 1.1 (10 – 30 % decrease in maximum diameter of target lesions). Where available, contrast-enhanced CT images were directly compared. Otherwise, nonenhanced CT images from the SPECT or PET components of the study were assessed, using metabolic uptake as a guide to follow the dominant lesions.

In patients included in the study due to symptomatic disease, benefit was defined as an improvement in tumour-related symptoms at 3 – 6 months after induction treatment. For those included due to previously progressive disease (on biochemical, molecular imaging or CT criteria), benefit was defined as stabilization or improvement of disease assessed at 3 – 6 months, and at 6 – 12 months after completion of induction treatment.

### Statistics

The OS curve was estimated for the total cohort of patients using the Kaplan–Meier product-limit method; point estimates and corresponding 95 % confidence intervals (95 % CI) were calculated for annual survival rates. The proportions of patients achieving biochemical, molecular imaging and CT responses were calculated separately with corresponding exact 95 % CIs for the subset of patients who were treated for previously progressive disease.

The following prognostic factors were investigated for association with both OS and treatment response: patient age, primary tumour site (pancreatic versus nonpancreatic), dominant site of disease (liver, bone, nodal or disseminated), number of tumour lesions (1 – 4, 5 – 20 or >20), size of the largest measurable diameter of the dominant lesion (<2 cm, 2 – 5 cm, >5 cm), percentage of whole-body retention, grade of tumour differentiation (*grade 1* Ki-67 index ≤2 %, *grade 2* Ki-67 index 3 – 20 %, *grade 3* Ki-67 index >20 %), and FDG avidity (*grade 1* uptake less than that of the liver, *grade 2* equal to that of the liver, *grade 3* slightly greater than that of the liver, *grade 4* markedly greater than that of the liver). Treatment-related parameters included planned number of cycles of ^177^Lu-octreotate (three versus four or more), number of prior cycles of ^111^In-octreotide (none, one or more), total cumulative activity of LuTate (in gigabecquerels), concurrent use of 5-FU chemotherapy and duration of treatment.

 Univariate associations with OS for the total patient cohort were investigated using Cox proportional hazards regression, where hazard ratios and associated 95 % CIs are reported. Tests for the association between response and OS were performed measuring OS from the start of treatment in order to facilitate comparison with previous work in this area, and also from 12 months after the end of treatment to remove bias associated with having a start date for OS that preceded the date at which response was measured. Univariate associations with each of biochemical, functional and CT responses for the subset of patients treated for previously progressive disease were investigated using binary logistic regression, where odds ratios and associated 95 % CIs are reported. Multivariate analysis was not performed because of the relatively small number of events and potential codependence of some of the analysed variables. Rather we sought to establish potential prognostic factors that might be further assessed in future prospective studies.

## Results

### Patient characteristics and treatment parameters

Of the 68 patients, 35 had a nonpancreatic primary, and 33 had a pancreatic primary tumour. A total of 251 cycles of LuTate therapy were administered (three cycles given to 26 patients, four cycles to 38 patients, five cycles to 3 patients, and six cycles to 1 patient). The median cumulative activity of the induction courses was 31 GBq (21 – 45.3 GBq) given over a median of 5 months (3 – 14 months). Concomitant 5-FU chemotherapy was administered to a majority of the cohort (63 patients). Five patients did not have concomitant 5-FU: one had previous 5-FU-related cardiotoxicity, two declined treatment despite being recommended to have 5-FU, and two were deemed unfit for chemotherapy. Of note, 27 patients also had previous high-activity ^111^In-octreotide treatment. Of these patients, three had received ^111^In-octreotide therapy (one to three cycles) 8 – 36 months before but they were eligible for subsequent LuTate therapy due to symptomatic or progressive disease. As part of induction PRCRT, 24 patients received high-activity ^111^In-octreotate therapy (20 patients had one cycle, 4 patients had two cycles) due to the presence of bony metastases in ten patients, a large tumour burden and potential flare/toxicity in four patients, and in ten patients for logistic reasons or limited availability of LuTate, particularly in the early stages when LuTate therapy first became available at our institution. Of note, eight patients received chemotherapy based on a Ki-67 index >10 %, or with concordant highly FDG avid disease before PRCRT. The majority had carboplatin/etoposide chemotherapy. One of these patients achieved a partial metabolic response after chemotherapy, but the remaining patients had either no response (six patients) or progression (one patient) on both molecular imaging and RECIST assessment. After subsequent LuTate induction treatment, the patient who progressed on chemotherapy continued to do so, suggesting refractory disease. However, all the seven other patients demonstrated a partial molecular imaging response and four of these patients also achieved a partial morphological response, suggesting that previous chemotherapy had minimal prognostic influence in these patients.

Of the 68 patients, 10 started treatment due to uncontrolled symptoms and 58 started treatment due to disease progression. The patient characteristics are summarized in Table 1.

**Table 1:** Patient baseline characteristics and treatment-related parameters for the total cohort of 68 patients

Characteristic	Disease progression (*N* = 58)	Uncontrolled symptoms (*N* = 10)	All patients (*N* = 68)
Age at first treatment (years)					
Median	55.5	57.5	56.0
Range	17 – 76	42 – 70	17 – 76
Primary site, *N* (%)					
Pancreatic NET	25 (43)	4 (40)	29 (43)
Small bowel	11 (19)	1 (10)	12 (18)
Large bowel	7 (12)	0 (0)	7 (10)
Lung	3 (5)	0 (0)	3 (4)
Gastrinoma	3 (5)	0 (0)	3 (4)
Glucagonoma	1 (2)	0 (0)	1 (1)
Thymus	0 (0)	1 (1)	1 (1)
Unknown	8 (14)	4 (40)	12 (18)
Nonpancreatic NET	29 (50)	6 (60)	35 (51)
Pancreatic NET	29 (50)	4 (40)	33 (49)
Dominant site of disease, *N* (%)					
Liver	36 (62)	6 (60)	42 (62)
Bone	1 (2)	0 (0)	1 (1)
Primary	4 (7)	1 (10)	5 (7)
Nodal	2 (3)	0 (0)	2 (3)
Disseminated	15 (26)	3 (30)	18 (26)
Number of lesions, *N* (%)					
1 – 4	4 (7)	2 (20)	6 (9)
5 – 20	36 (62)	6 (60)	42 (62)
>20	18 (31)	2 (20)	20 (29)
Size of dominant lesion (cm), *N* (%)					
<2	1 (2)	0 (0)	1 (1)
2 – 5	32 (55)	7 (70)	39 (57)
>5	24 (41)	3 (30)	27 (40)
Unknown	1 (2)	0 (0)	1 (1)
Whole-body retention (%)					
Median	18.1	16.0	
Range	5.8 – 40.9	5.6 – 40.9	
Grade of tumour differentiation, *N* (%)					
1 (Ki-67 index < 3 %)	7 (12)	2 (20)	9 (13)
2 (Ki-67 index 3 – 20 %)	26 (45)	4 (40)	30 (44)
3 (Ki-67 index > 20 %)	0 (0)	0 (0)	0 (0)
Unknown	25 (43)	4 (40)	29 (43)
FDG avidity grade before treatment, *N* (%)					
0 (no uptake)	2 (3)	0 (0)	2 (3)
1 (< liver)	0 (0)	0 (0)	0 (0)
2 (= liver)	1 (2)	0 (0)	1 (1)
3 (slightly > liver)	11 (19)	2 (20)	13 (19)
4 (markedly > liver)	10 (17)	1 (10)	11 (16)
Unknown	34 (59)	7 (70)	41 (60)
Cumulative LuTate activity (GBq)					
Median	30.9	31.0	
Range	21.0 – 45.3	21.0 – 45.3	
Treatment duration (weeks)					
Median	20.0	21.0	
Range	12 – 62	12 – 62	

### Overall survival

**Fig. 1:**
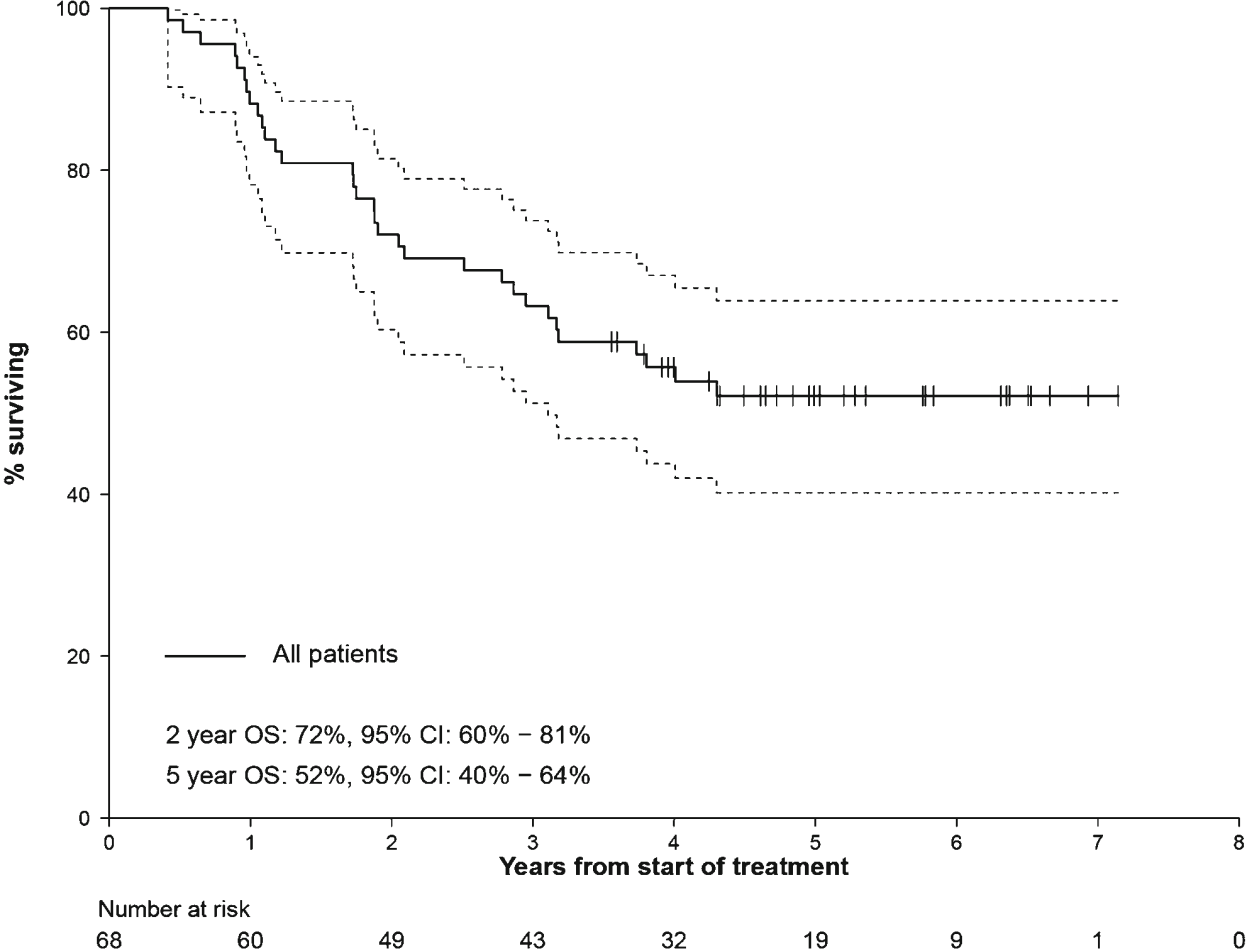
Estimated Kaplan–Meier curve for OS from the start of the first cycle of treatment

Of the 68 patients, 32 died before the close date. The observed annual OS rates were as follows: 1 year 88 % (95 % CI 78 – 94 %), 2 years 72 % (95 % CI 60 – 81 %), 3 years 63 % (95 % CI 51 – 74 %), 4 years 56 % (95 % CI 44 – 67 %), and 5 years 52 % (95 % CI 40 – 64 %). Median OS was not estimable despite a median follow-up period of 60 months (Fig. 1).

### Overall response at 3 – 6 months after treatment

#### Uncontrolled symptoms

Of the 68 patients, 10 were treated for tumour-related symptomatic disease. At 3 months after treatment, nine patients reported symptomatic response. At 6 months, seven patients (70 %) reported continuing benefit from treatment. However, two patients had redeveloped increasing pain related to further progressive disease despite treatment, and one patient reported worsening carcinoid hormonal symptoms despite a good initial symptomatic response. Most patients reported improved symptoms with reduced flushing, diarrhoea, soft-tissue and bone pain as well as weight gain.

#### Previous progressive disease

Of the 68 patients, 58 were treated for disease progression. A high proportion of these patients (44, 76 %) received benefit from PRRT resulting in either stabilization or improvement on imaging of previously progressive disease. However, one patient died at 8 months despite a good early response. Of the 58 patients, 14 (24 %) had no clear benefit from treatment with early disease progression, and 12 of these patients died within 12 months of the last treatment.

### Objective response at 6 – 12 months after treatment in patients with previous progressive disease

#### Biochemical (CgA) response

**Fig. 2:**
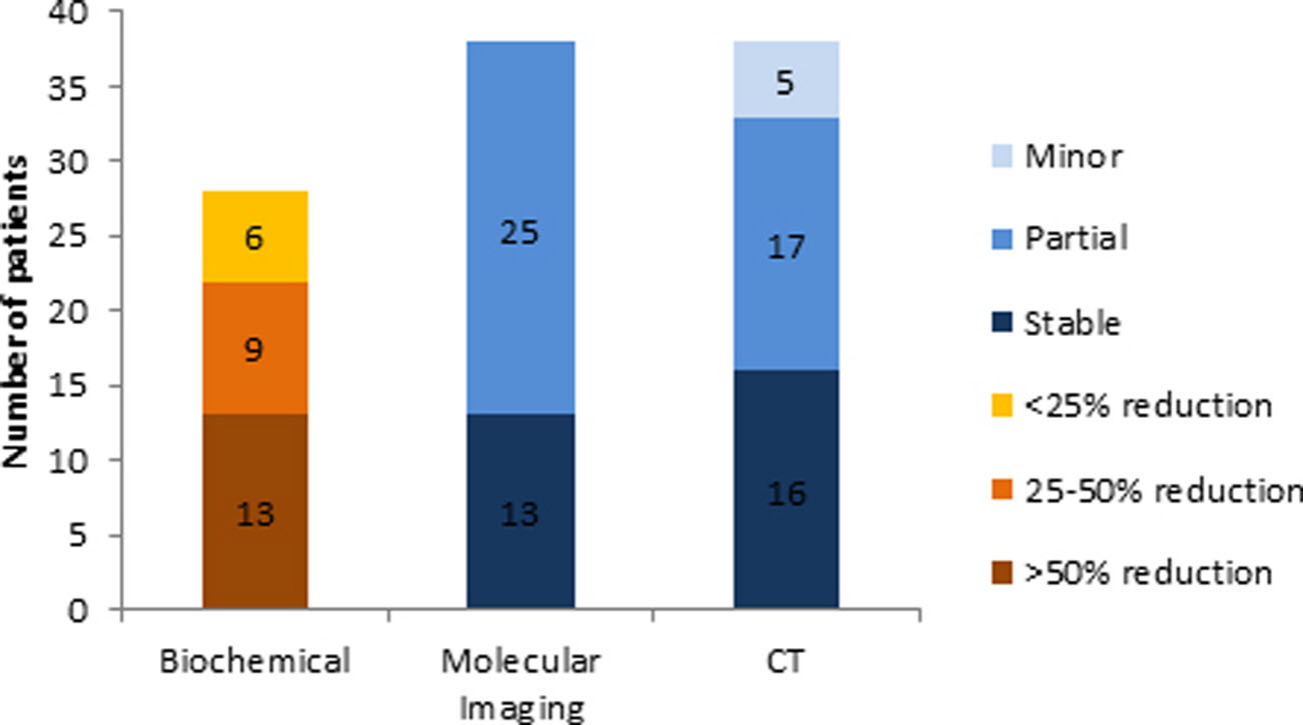
Biochemical, molecular imaging and CT responses 6 – 12 months after the last induction cycle of LuTate therapy in patients with previously progressive disease. The overall biochemical, molecular imaging and CT response rates were 56 %, 67 % and 68 %, respectively

Of the 58 patients with progressive disease, 2 had no follow-up CgA results available, and 6 had a very low baseline level (<30 U/L); therefore these patients were excluded. Hence, 50 patients were for included in the analysis, and 28 of these (56 %) achieved a biochemical response as defined as a decrease from the baseline level (Fig. 2).Of these, 13/28 had >50 % reduction from baseline, 9/28 had 25 – 50 % reduction from baseline, and 6/28 had <25 % reduction from baseline. 9/50 patients (18 %) had documented CgA progression over baseline level. However, three of these patients only had a mild relative increase of <25 % over baseline, and whilst counted as progression, may have represented a slowing of disease.

#### Molecular imaging response

One of 58 patients had no available follow-up; hence 57 patients were evaluated. Overall 38 of 57 patients (67 %) had benefit (95 % CI 53 – 79 %): 13 of 57 (23 %) had stabilization of previously progressive disease, and 25 of 57 (44 %) achieved a partial response (Fig. 2). Only 3 of 57 patients had progressive disease, and 3 had a mixed response but were still assessable at 12 months.

#### CT response

Two of 58 patients had no CT-evaluable lesions; hence 56 patients were evaluated. Overall 38 of 56 patients (68 %) had a response (95 % CI 54 – 80 %): 16 of 56 (29 %) had stable disease, 17 of 56 (30 %) had a partial response, and 5 of 56 (9 %) had a minor response (Fig. 2). A further 4 of 56 patients had disease progression and 1 had a mixed response. Of these patients, 13 died of disease progression within 12 months. Of the 68 patients, 11 had a further response beyond 12 months after the last induction treatment cycle, and before any maintenance PRRT. One of these patients had achieved a complete molecular imaging and CT response at 3 years after the last induction cycle. Another patient had the tumour completely resected as a result of a significant response to induction treatment.

Of the 68 patients, 44 (68 %) received subsequent LuTate maintenance therapy: 9 patients had one cycle, 16 patients two cycles, 9 patients three cycles, 5 patients four cycles, 2 patients five cycles, and 3 patients six cycles. Of these patients, 17 had no documented disease progression from after induction treatment to the time of this report, but were considered at high risk of progression. Eight patients with subsequent progression after initial induction LuTate treatment had achieved further stabilization or regression of disease with maintenance therapy. Despite an initial favourable response to induction cycles, 19 patients developed subsequent disease progression despite further maintenance treatments.

### Prognostic/predictive factors associated with overall survival and objective response

#### Overall survival

**Fig. 3:**
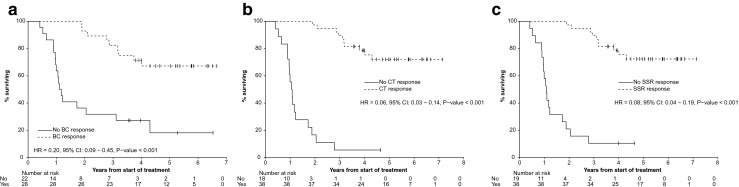
Estimated Kaplan–Meier OS curve associated with **a** biochemical, **b** CT, and **c** molecular imaging responses from the start of the first cycle of treatment

Patients with a disseminated pattern of disease had a significantly increased risk of death relative to patients in whom the dominant site of disease was the liver. For every unit increase in percentage of whole-body retention, the risk of death increased providing potential prognostic stratification at the commencement of treatment. A biochemical, functional molecular imaging and CT imaging response at 6 – 12 months was also significantly associated with improved OS. Imaging responses (functional imaging and CT) yield strongly significant results when measured from 6 to 12 months after treatment (Fig. 3, Table 2).

**Table 2:** Factors associated with overall survival

Factor	Hazard ratio	95 % CI	*P* value
Dominant site of disease			
Liver			0.03
Bone	NE	–	
Primary	0.50	0.07 – 3.74	
Nodal	1.33	0.18 – 9.99	
Disseminated	2.78	1.34 – 5.74	
Whole-body retention			
≤16 %			
>16 %	2.07	1.02 – 4.21	0.04
As continuous variable	1.06	1.02 – 1.10	0.002
Response measured from start of treatment			
Biochemical response	0.20	0.09 – 0.45	<0.001
Molecular imaging response	0.08	0.04 – 0.19	<0.001
CT response	0.06	0.03 – 0.14	<0.001
Response measured from 6 to 12 months after treatment response assessment			
Biochemical response	0.59	0.18 – 1.9	0.382
Molecular imaging response	0.2	0.06 – 0.64	0.003
CT response	0.13	0.04 – 0.2	<0.01

*NE* not estimable (when no deaths were observed in one of the groups being compared)

#### Biochemical response

Patients with a primary pancreatic NET (compared to nonpancreatic), a disseminated pattern, and the dominant lesion >5 cm in size were statistically significantly less likely to achieve a biochemical response (Table 3).

**Table 3:** Factors associated with objective response (biochemical, molecular imaging, CT) at 6 – 12 months after induction treatment in 58 patients with previously progressive disease

	Factor	Biochemical response				Molecular imaging response				CT response			
		%	Odds ratio	95 % CI	*P* value	%	Odds ratio	95 % CI	*P* value	%	Odds ratio	95 % CI	*P* value
Primary tumour site	Nonpancreatic	74			0.02*	82			0.01*	81			0.03*
	Pancreatic	41	0.24	0.07 – 0.81		52	0.23	0.07 – 0.78		55	0.28	0.08 – 0.94	
Dominant disease site	Liver	66			0.02*	71			0.24	74			0.16
	Bone					100	NE						
	Primary	33	0.26	0.02 – 3.18		75	1.20	0.11 – 12.9		75	1.04	0.10 – 11.3	
	Nodal	100	NE			100	NE			100	NE		
	Disseminated	20	0.13	0.02 – 0.71		47	0.35	0.10 – 1.22		47	0.30	0.09 – 1.07	
Size of dominant lesion	≤5 cm	70			0.01*	76			0.07	79			0.04*
	>5 cm	35	0.23	0.07 – 0.77		52	0.35	0.11 – 1.09		52	0.29	0.09 – 0.95	
Concurrent 5-FU chemotherapy	No cycles	40				0				0			
	One or more cycles	57	1.30	0.17 – 10.0	0.80	70	NE		0.008*	72	NE		0.007*

*P < 0.05

*NE* not estimable (when no deaths were observed in one of the groups being compared)

#### Functional imaging response

Patients with a primary pancreatic NET were significantly less likely to achieve a molecular imaging response, while patients who underwent concurrent 5-FU chemotherapy were more likely to achieve a molecular imaging response even though the number of patients not receiving chemotherapy (*n* = 5) was low (Table 3).

#### CT response

Significant univariate associations were found for primary tumour site, size of dominant lesion <5 cm and concurrent use of 5-FU chemotherapy (Table 3 and Supplementary table online). A subgroup of patients who had partial and minor CT response (excluding stable disease) were also analysed, and significant positive associations were found for the number of tumour lesions (four or fewer), number of LuTate cycles (four or more), total cumulative activity, and concurrent 5-FU chemotherapy.

## Discussion

LuTate PRRT has been used to treat patients with inoperable metastatic NET with high SSTR expression, but comparison of toxicity and outcomes have often been difficult due to heterogeneous patient populations, variable study designs, and different response assessments. Importantly, some studies showing high rates of stable disease have not required disease progression prior to PRRT therapy. In contrast to the other published series, however, our study had a long follow-up assessment for both objective response and OS, comprising a balanced proportion of primary tumour site (pancreatic versus nonpancreatic), and is, we believe, the largest study utilizing LuTate with 5-FU chemoradionuclide therapy. The use of PRCRT based on compassionate grounds involved application of strict inclusion criteria limiting treatment to patients with symptomatic or progressive disease uncontrolled by conventional therapy. In fact, only a minority of our cohort were treated for uncontrolled symptoms, possibly because of an aggressive policy of dose escalation of somatostatin analogues if tolerated by the patient. Despite this approach, up to 70 % of our cohort reported improvement beyond 6 months after PRCRT. This also supports the findings of another study including 50 patients evaluated for quality of life that showed a significant improvement in global health status at 6 weeks after the last ^177^Lu-octreotate cycle [14]. A study in 265 patients by Khan et al. also showed an improvement in quality of life and Karnofsky performance score [15]. The majority of our patients were treated for disease progression within 12 months, and this possibly explains the significant proportion of grade 2 tumours in our series (Table 4).

**Table 4:** Comparison of studies with ^177^Lu-octreotate therapy

Reference	No. of patients	Treatment criteria	Primary site	Disease grade	Cumulative activity (GBq)	Reported response	Response assessed	Median follow -up	Overall survival
[9]	310	Tumour uptake > liver uptake on Octreoscan, no previous PRRT, progression in 133 patients	Pancreatic 29 %, carcinoid 61 %, unknown 10 %	Not specified	27.8 – 29.6	46 % complete response, partial response and minor response on CT (SWOG criteria)	3 months after last therapy cycle	19 months	Median 46 months
[18]	21	Disseminated NET with high SSTR expression, progression not specified	Pancreatic 14 %, carcinoid 86 %	Grade 1 (12 patients), grade 2 (8 patients)	Maximal tolerated absorbed dose of 27 Gy to the kidneys	Twelve patients evaluable, partial response 17 %, minor response 25 %, stable disease 42 % (RECIST)	At least 10 months	Not available	Not available
[19]	33	Positive Octreoscan, progression on CT/MRI	Pancreatic 30 %, carcinoid 52 %, unknown 18 %	Cellular proliferation rate <5 % when specified	Intended four cycles of 7.8 GBq with capecitabine	Partial response 24 %, stable disease 70 % (RECIST 1.1)	6 months	16 months (5 – 33 months)	88 % at 2 years, median not reached
[20]	51	Unresectable or metastatic NET, positive Octreoscan (progression in 39/49 assessable patients)	Pancreatic 27 %, carcinoid 67 %, unknown 6 %	Ki-67 index (available in 29 patients) of <20 % in 25/29 patients, >20 % in 4 patients	Group 1 median 26.4, group 2 median 25.2	Complete response + partial response 32.6 % (RECIST)	After last therapy	29 months (4 – 66 months)	68 % at 36 months, median not reached
Current study (2013)	68	Progressive disease (85 %) or uncontrolled symptoms despite conventional therapy for high SSTR disease	Pancreatic 49 %, carcinoid 33 %, unknown 18 %	Grade 1, grade 2 (up to 44 %)	Median 31, majority four cycles with 5-FU	Biochemical 56 %, molecular imaging 67 %, CT 68 % (stable disease or regression) (RECIST 1.1)	Up to 12 months after last therapy cycle	60 months (5 – 86 months)	52 % at 5 years, median not reached

**Fig. 4:**
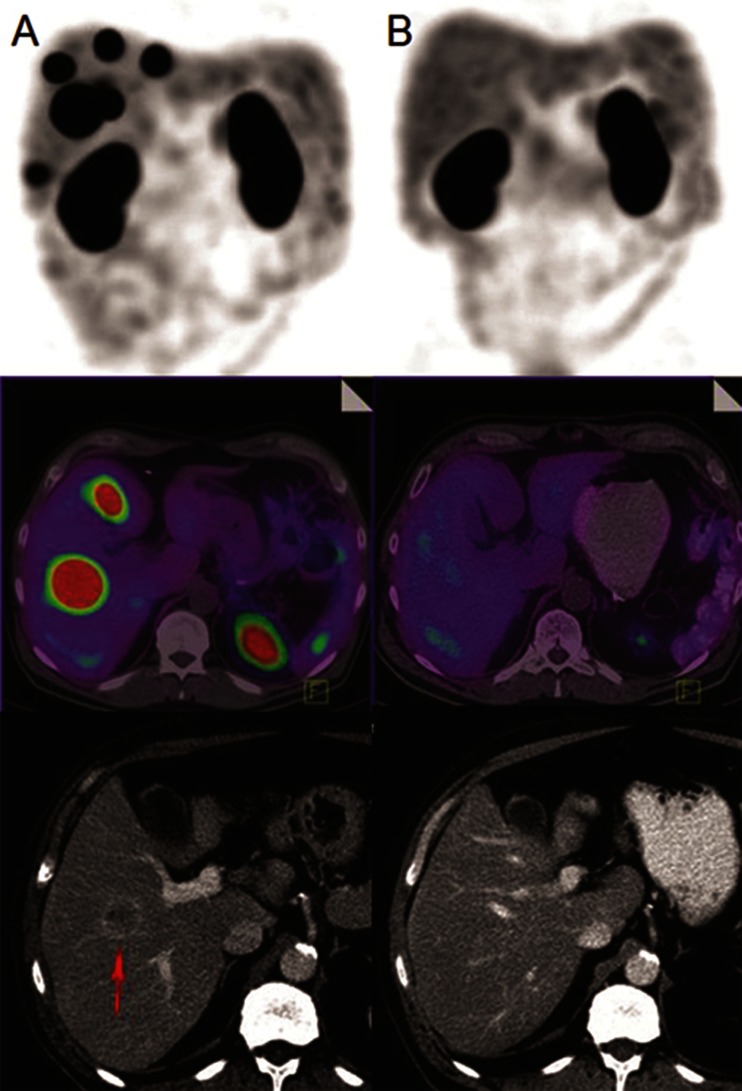
A 63-year-old patient with previously resected primary pancreatic NET referred for LuTate therapy due to progressive disease on CT in relation to multiple SSTR-positive liver metastases. *Top* Maximum intensity projection images. *Centre* Fused transaxial SPECT/CT images. *Bottom* Contrast-enhanced CT images. **a** After the first cycle of LuTate therapy at treatment baseline, the images show high SSTR-expressing liver metastases (*red arrow* most dominant lesion). **b** Complete scintigraphic and anatomical CT response to treatment approximately 3 years after induction LuTate therapy without any intervening treatments and despite only a partial early response. This patient remained disease-free at the time of this report

Among the majority of this cohort who were treated for progressive disease, a high proportion (76 %) received benefit based on disease stabilization or regression at the early (3 – 6 months) assessment after completion of induction treatment, and a high percentage continued to demonstrate benefit at 6 – 12 months after treatment. Given that the median duration of induction treatment was 5 months, the initial response assessment was typically 8 – 12 months following the start of treatment, an interval over which most patients had previously progressed. Both CT and functional molecular imaging showed a similar rate of disease stabilization or regression (68 % versus 67 %), although molecular imaging was more often associated with regression (44 % versus 29 %; all partial response). Of note, 9 % of patients also had a minor CT response. One patient who responded significantly on both CT and functional imaging underwent complete resection of residual disease, which had initially been assessed to be inoperable, supporting the role of PRCRT. Thus, PRCRT may render a small proportion of inoperable NET resectable [16]. In addition, 16 % of patients also demonstrated further response beyond 12 months after completion of induction treatment, including one patient who achieved a complete response on molecular imaging and CT criteria at 3 years after the induction cycle without any additional therapy, suggesting an ongoing and potentially delayed therapeutic effect in some patients (Fig. 4). This phenomenon has also been described by Kwekkeboom et al. [9]. The overall findings are at least comparable to those of other series with objective partial response rates of 17 – 28 % [17–20]. The largest series showed a CT partial response of 28 % and a minor response of 16 % [9]. However, such a high rate of response including disease stabilization in this cohort is a particularly encouraging result given that our patients were treated in the setting of progressive disease with otherwise limited therapeutic options.

Several factors were found to be significantly associated with an objective response. These included primary tumour site, dominant site of disease, size of dominant lesion, and the use of concurrent 5-FU chemotherapy. Although there was no statistically significant association with OS, patients with a nonpancreatic primary tumour seemed more likely to have biochemical, molecular imaging and CT responses, suggesting that disease from a primary pancreatic tumour is more resistant and difficult to control, and hence a more aggressive therapeutic approach/regimen may be warranted. Patients with dominant liver disease compared to those with a disseminated pattern are more likely to have biochemical response. Patients with a dominant lesion size of <5 cm are significantly more likely to achieve molecular imaging and CT response. Therefore, using more powerful radiopharmaceuticals such as ^90^Y, which has a longer beta radiation path length, may be more efficacious in treating larger lesions. Although few patients did not receive 5-FU chemotherapy, the use of 5-FU chemotherapy was found to be strongly associated with molecular imaging and CT response in this cohort. Favourable response rates without associated increases in toxicity have also been reported from a recent study using LuTate combined with capecitabine, the oral form of 5-FU chemotherapy [19]. A previous study from our institution showed no significant additional toxicity when 5-FU was given with high-activity ^111^In-octreotide therapy [6] and in a patient population overlapping with that of the current study also showed that administration of LuTate after previous high-dose ^111^In-octreotide therapy is safe [10].

Of interest, factors associated with tumour shrinkage on CT included fewer lesions, more LuTate cycles, and higher total cumulative activity. This suggests a possible dose–response effect and warrants investigation of personalized dosimetry. Other prognostic factors for predicting tumour remission observed in previous studies include high uptake on diagnostic Octreoscan and a Karnofsky performance score of >70 [9]. Both these factors tend to be associated with lower tumour burden. Another study also suggested that a low tumour burden and a low proliferation index are prognostic factors for long progression-free survival [21].

One of the major advantages of this study was the long follow-up, allowing determination of OS rates at 2 and 5 years (which were 72 % and 52 %, respectively), with median survival not estimable despite this. Our results are at least comparable, if not superior, to those of the largest published series which showed a median OS of 46 months from the start of treatment [9]. Patients with a disseminated pattern and large disease burden had a significantly increased risk of death. Most importantly, biochemical, molecular or CT stabilization or regression within 12 months was significantly associated with longer OS. Our results support those of a prior study indicating that tumour shrinkage may not be related to survival [20] and also indicate that molecular imaging response provides a potential imaging biomarker of response given its association with improved survival. The predictive value of molecular imaging response was also maintained when survival was estimated from the date of the scan. It may therefore provide an alternative assessment tool to CT anatomical response. CT responses using RECIST criteria alone may not accurately assess NET liver lesions in the posttreatment setting due to variable contrast enhancement and necrosis leading to pseudoprogression. Having molecular imaging to characterize lesions is pivotal to selection of target lesions and their follow-up.

**Fig. 5:**
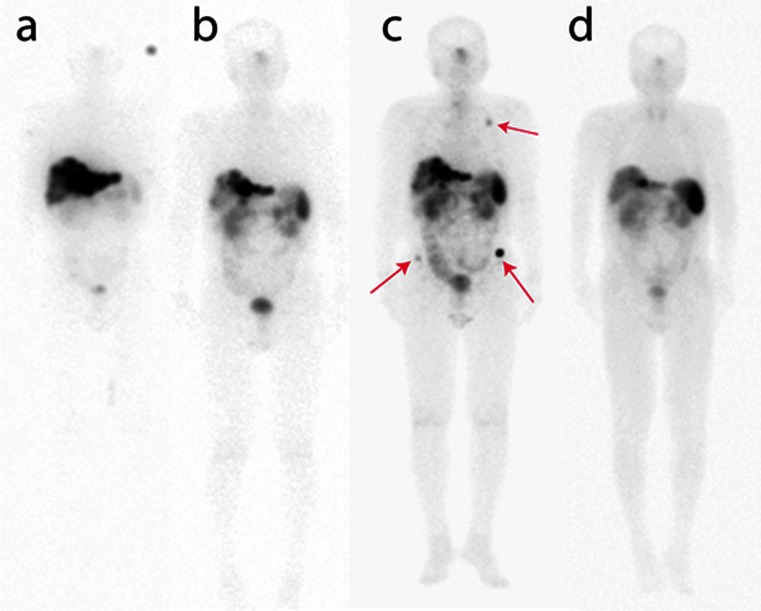
A 72-year-old patient with a primary of the pancreatic tail with liver metastases (grade 2 tumour, Ki-67 index 10 – 20 %) and progressive disease despite carboplatin/etoposide chemotherapy. **a** After the first cycle of LuTate, whole-body planar images show dominant high uptake in multiple liver lesions. **b** ^111^In-Octreotate whole-body planar image 15 months after completion of induction therapy shows a significant scintigraphic and CT response (latter not shown), with significant reduction of CgA levels from baseline (460 to 64 U/L). **c** Restaging ^111^In-Octreotate planar image at 27 months after completion shows progression of liver metastases and new small volume skeletal metastases (ribs, pelvis *red arrows*). These imaging findings were accompanied by symptoms of lethargy and weight loss. Given a previous favourable response to therapy, a further cycle of maintenance LuTate treatment (with 5-FU chemotherapy) was given. **d** Restaging ^111^In-octreotate images 14 months after maintenance therapy show a dramatic further response to treatment with marked regression of liver disease and resolution of multifocal bone disease. This was accompanied by significant improvement in symptoms

Our regular maintenance treatment protocol may have contributed to the overall good survival. The majority of patients remained stable without progression on maintenance suggesting a tumoristatic effect. However, further stabilization or regression was also seen in patients with subsequent progression after an initial response (Fig. 5). Two studies have also shown that the use of LuTate as salvage treatment is effective and safe in patients with progressive disease after initial benefit from induction treatment [22, 23].

This study was designed to assess the predictors of response and long-term survival of progressive NET treated with PRCRT; hence toxicity was not evaluated in this cohort but was the subject of previous studies in overlapping patient cohorts. We have previously reported the lack of significant short-term nephrotoxicity or myelotoxicity of LuTate PRCRT [10]. Similarly, we also recently reported the lack of long-term nephrotoxicity with LuTate PRCRT [24]. One patient in this cohort with progressive NET who received a total of seven cycles of PRCRT (cumulative activity 56.4 GBq) over 4 years but in the setting of previous chemotherapy treatments was subsequently diagnosed with acute myeloid leukaemia with underlying myelodysplastic changes despite a good initial response from PRCRT. Another patient was diagnosed with myelodysplastic syndrome (MDS) after five cycles of PRCRT (37.9 GBq), but this was considered not to be treatment-related given preexisting thrombocytopaenia at the time of the first PRRT and with a translocation not typical of radiation-induced MDS. Serious late adverse events probably caused by PRRT are rare, with MDS occurring in approximately 1 % of patients in the largest series [9].

This analysis did, however, have recognized limitations related to the retrospective nature of the study that potentially confounded response and prognostic assessment. Being a compassionate treatment, the treatment strategy was personalized rather than protocol-driven. Indeed, there has been an evolution in our treatment approach over the past 5 years. Towards the end of the period covered by this cohort, we began to treat more rapidly progressive disease more using higher administered activities, decreased intervals between cycles, or additional induction LuTate cycles. We have also attempted to deliver higher cumulative activities to patients with larger disease burdens. Whether these changes will improve patient outcomes requires further assessment. However, there was no significant association between the year of starting PRCRT and OS. Further, since the Ki-67 proliferative index was not routinely determined particularly in patients who were recruited early in this cohort, incomplete data restricted assessment of this parameter as a prognostic factor. Finally, many patients were comanaged by clinicians outside our own institution, and a uniform follow-up approach was logistically challenging. To prevent unnecessary duplication of diagnostic CT performed elsewhere, triple phase CT was not routinely repeated but was performed if lesions could not be adequately assessed by non-contrast CT guided by the functional imaging data. Almost all patients regularly returned to our institution for molecular imaging follow-up with SPECT/CT or PET/CT enabling adequate comparative RECIST measurements.

Given the overall encouraging results in terms of response and OS in our study and other previous studies, LuTate therapy can be recommended for patients with symptomatic or progressive grade 1 or grade 2 NET who have failed or are unsuitable for alternative therapies. Combining with 5-FU chemotherapy whenever clinically appropriate may also provide synergistic benefit. With increasing recognition of the heterogeneous phenotypes of NET and expanding multimodality treatment options, we believe that a more individualized approach should be adopted to further maximize therapeutic benefit. In particular, our results suggest that patients with metastatic disease from primary pancreatic tumour and those with larger dominant lesion size (>5 cm) may require a more aggressive treatment regimen. Options would include combining LuTate with ^90^Y PRRT [25–27]. Surgical debulking following response from induction PRCRT could be considered where appropriate. Based on our recent study suggesting that tumour sequestration is a major factor leading to a ‘sink effect’ that decreases activity concentration in healthy organs such as the kidney [28], we hypothesize that giving higher administered activity per cycle particularly in those with larger disease burden may allow greater radiation dose to individual lesions and increased therapeutic index.

Given the favourable overall results in this series and recent analysis of our results in patients with a poorer prognosis based on FDG PET/CT (manuscript in preparation), we have increasingly used PRCRT as first-line treatment in patients with progressive tumours who remain amenable to PRCRT as a result of adequate SSTR expression at all known sites of disease irrespective of Ki-67 (including grade 2 NET and grade 3 neuroendocrine carcinoma). In addition, combining LuTate with other concomitant chemotherapy agents to further increase efficacy should also be considered. Recent studies have shown that capecitabine and temozolomide are highly active and well tolerated in well-differentiated metastatic pancreatic NET [29, 30]. A recent study combining this regimen with LuTate has shown promising results [31], and therefore may be considered in patients with metastatic grade 2 NET or grade 3 neuroendocrine carcinoma of the pancreas.

### Conclusion

This study suggests that LuTate PRCRT results in therapeutic benefit in a high proportion of patients with uncontrolled symptoms or previously progressive NET with high SSTR expression despite conventional treatment. Patients achieving objective biochemical, molecular imaging or CT responses within 12 months, and those with a limited and lower disease burden tended to show a longer OS. Patients with a primary pancreatic tumour, larger lesions and higher disease burden may need a more aggressive treatment approach.

## Electronic supplementary material

Below is the link to the electronic supplementary material.

ESM 1 (DOCX 36 kb)

## References

[1] Rindi G (2010). The ENETS guidelines: the new TNM classification system. Tumori.

[2] Zaknun JJ, Bodei L, Mueller-Brand J, Pavel ME, Baum RP, Horsch D (2013). The joint IAEA, EANM, and SNMMI practical guidance on peptide receptor radionuclide therapy (PRRNT) in neuroendocrine tumours. Eur J Nucl Med Mol Imaging.

[3] Imhof A, Brunner P, Marincek N, Briel M, Schindler C, Rasch H (2011). Response, survival, and long-term toxicity after therapy with the radiolabeled somatostatin analogue [90Y-DOTA]-TOC in metastasized neuroendocrine cancers. J Clin Oncol.

[4] Bodei L, Cremonesi M, Grana CM, Chinol M, Baio SM, Severi S (2012). Yttrium-labelled peptides for therapy of NET. Eur J Nucl Med Mol Imaging.

[5] Teunissen JJ, Kwekkeboom DJ, Valkema R, Krenning EP (2011). Nuclear medicine techniques for the imaging and treatment of neuroendocrine tumours. Endocr Relat Cancer.

[6] Kong G, Johnston V, Ramdave S, Lau E, Rischin D, Hicks RJ (2009). High-administered activity In-111 octreotide therapy with concomitant radiosensitizing 5FU chemotherapy for treatment of neuroendocrine tumors: preliminary experience. Cancer Biother Radiopharm.

[7] Pavel M, Kidd M, Modlin I (2013). Systemic therapeutic options for carcinoid. Semin Oncol.

[8] Toumpanakis C, Caplin ME (2013). Update on the role of somatostatin analogs for the treatment of patients with gastroenteropancreatic neuroendocrine tumors. Semin Oncol.

[9] Kwekkeboom DJ, de Herder WW, Kam BL, van Eijck CH, van Essen M, Kooij PP (2008). Treatment with the radiolabeled somatostatin analog [177Lu-DOTA 0, Tyr3]octreotate: toxicity, efficacy, and survival. J Clin Oncol.

[10] Hubble D, Kong G, Michael M, Johnson V, Ramdave S, Hicks RJ (2010). 177Lu-octreotate, alone or with radiosensitising chemotherapy, is safe in neuroendocrine tumour patients previously treated with high-activity 111In-octreotide. Eur J Nucl Med Mol Imaging.

[11] Hofman MS, Hicks RJ (2012). Changing paradigms with molecular imaging of neuroendocrine tumors. Discov Med.

[12] Wahl RL, Jacene H, Kasamon Y, Lodge MA (2009). From RECIST to PERCIST: evolving considerations for PET response criteria in solid tumors. J Nucl Med.

[13] Hicks RJ (2005). The role of PET in monitoring therapy. Cancer Imaging.

[14] Teunissen JJ, Kwekkeboom DJ, Krenning EP (2004). Quality of life in patients with gastroenteropancreatic tumors treated with [177Lu-DOTA0, Tyr3]octreotate. J Clin Oncol.

[15] Khan S, Krenning EP, van Essen M, Kam BL, Teunissen JJ, Kwekkeboom DJ (2011). Quality of life in 265 patients with gastroenteropancreatic or bronchial neuroendocrine tumors treated with [177Lu-DOTA0, Tyr3]octreotate. J Nucl Med.

[16] Barber TW, Hofman MS, Thomson BN, Hicks RJ (2012). The potential for induction peptide receptor chemoradionuclide therapy to render inoperable pancreatic and duodenal neuroendocrine tumours resectable. Eur J Surg Oncol.

[17] Kam BL, Teunissen JJ, Krenning EP, de Herder WW, Khan S, van Vliet EI (2012). Lutetium-labelled peptides for therapy of neuroendocrine tumours. Eur J Nucl Med Mol Imaging.

[18] Garkavij M, Nickel M, Sjogreen-Gleisner K, Ljungberg M, Ohlsson T, Wingardh K (2010). 177Lu-[DOTA0, Tyr3] octreotate therapy in patients with disseminated neuroendocrine tumors: analysis of dosimetry with impact on future therapeutic strategy. Cancer.

[19] Claringbold PG, Brayshaw PA, Price RA, Turner JH (2011). Phase II study of radiopeptide 177Lu-octreotate and capecitabine therapy of progressive disseminated neuroendocrine tumours. Eur J Nucl Med Mol Imaging.

[20] Bodei L, Cremonesi M, Grana CM, Fazio N, Iodice S, Baio SM (2011). Peptide receptor radionuclide therapy with (177)Lu-DOTATATE: the IEO phase I-II study. Eur J Nucl Med Mol Imaging.

[21] Campana D, Capurso G, Partelli S, Nori F, Panzuto F, Tamburrino D (2013). Radiolabelled somatostatin analogue treatment in gastroenteropancreatic neuroendocrine tumours: factors associated with response and suggestions for therapeutic sequence. Eur J Nucl Med Mol Imaging.

[22] van Essen M, Krenning EP, Kam BL, de Herder WW, Feelders RA, Kwekkeboom DJ (2010). Salvage therapy with (177)Lu-octreotate in patients with bronchial and gastroenteropancreatic neuroendocrine tumors. J Nucl Med.

[23] Sabet A, Haslerud T, Pape UF, Sabet A, Ahmadzadehfar H, Grunwald F (2014). Outcome and toxicity of salvage therapy with Lu-octreotate in patients with metastatic gastroenteropancreatic neuroendocrine tumours. Eur J Nucl Med Mol Imaging.

[24] Kashyap R, Jackson P, Hofman MS, Eu P, Beauregard JM, Zannino D (2013). Rapid blood clearance and lack of long-term renal toxicity of Lu-DOTATATE enables shortening of renoprotective amino acid infusion. Eur J Nucl Med Mol Imaging.

[25] Kunikowska J, Krolicki L, Hubalewska-Dydejczyk A, Mikolajczak R, Sowa-Staszczak A, Pawlak D (2011). Clinical results of radionuclide therapy of neuroendocrine tumours with 90Y-DOTATATE and tandem 90Y/177Lu-DOTATATE: which is a better therapy option?. Eur J Nucl Med Mol Imaging.

[26] Villard L, Romer A, Marincek N, Brunner P, Koller MT, Schindler C (2012). Cohort study of somatostatin-based radiopeptide therapy with [(90)Y-DOTA]-TOC versus [(90)Y-DOTA]-TOC plus [(177)Lu-DOTA]-TOC in neuroendocrine cancers. J Clin Oncol.

[27] Pach D, Sowa-Staszczak A, Kunikowska J, Krolicki L, Trofimiuk M, Stefanska A (2012). Repeated cycles of peptide receptor radionuclide therapy (PRRT) – results and side-effects of the radioisotope 90Y-DOTA TATE, 177Lu-DOTA TATE or 90Y/177Lu-DOTA TATE therapy in patients with disseminated NET. Radiother Oncol.

[28] Beauregard JM, Hofman MS, Kong G, Hicks RJ (2012). The tumour sink effect on the biodistribution of 68Ga-DOTA-octreotate: implications for peptide receptor radionuclide therapy. Eur J Nucl Med Mol Imaging.

[29] Fine RL, Gulati AP, Krantz BA, Moss RA, Schreibman S, Tsushima DA (2013). Capecitabine and temozolomide (CAPTEM) for metastatic, well-differentiated neuroendocrine cancers: the Pancreas Center at Columbia University experience. Cancer Chemother Pharmacol.

[30] Strosberg JR, Fine RL, Choi J, Nasir A, Coppola D, Chen DT (2011). First-line chemotherapy with capecitabine and temozolomide in patients with metastatic pancreatic endocrine carcinomas. Cancer.

[31] Claringbold PG, Price RA, Turner JH (2012). Phase I–II study of radiopeptide 177Lu-octreotate in combination with capecitabine and temozolomide in advanced low-grade neuroendocrine tumors. Cancer Biother Radiopharm.

