# Supplementary material for: Assessment of predictors of response and long-term survival of patients with neuroendocrine tumour treated with peptide receptor chemoradionuclide therapy (PRCRT)
Source: Eur J Nucl Med Mol Imaging. 2014 May 21;41(10):1831–44. doi: 10.1007/s00259-014-2788-5 (PMC4159597; doi:10.1007/s00259-014-2788-5)
Supplement: Supplementary file 1 — (DOCX 36 kb) [file 259_2014_2788_MOESM1_ESM.docx]

|  | **Factor** | **Biochemical Response** | | | | | **Molecular Imaging Response** | | | | **CT Response** | | | | **Overall Survival** | |  | | |  |
| --- | --- | --- | --- | --- | --- | --- | --- | --- | --- | --- | --- | --- | --- | --- | --- | --- | --- | --- | --- | --- |
|  |  | **%** | **OR** | **95% CI** | ***P*-value** | | **%** | **OR** | **95% CI** | ***P*-value** | **%** | **OR** | **95% CI** | ***P*-value** | **HR** | **95%CI** | | ***P-*value** | | |
| **Age (years) at first treatment** | ≤ 56 | 52% |  |  |  | | 57% |  |  |  | 59% |  |  |  |  |  | | |  | |
|  | > 56 | 62% | 1.52 | (0.48, 4.76) | | 0.47 | 78% | 2.68 | (0.84, 8.53) | 0.09 | 78% | 2.47 | (0.77, 7.96) | 0.12 | 0.94 | (0.47, 1.89) | | 0.87 | | |
|  |  |  |  |  | |  |  |  |  |  |  |  |  |  |  |  | |  | | |
| **Primary tumour site** | Non-pancreatic | 74% |  |  | |  | 82% |  |  |  | 81% |  |  |  |  |  | |  | | |
|  | Pancreatic | 41% | 0.24 | (0.07, 0.81) | | **0.02** | 52% | 0.23 | (0.07, 0.78) | **0.01** | 55% | 0.28 | (0.08, 0.94) | **0.03** | 1.69 | (0.84, 3.41) | | 0.14 | | |
|  |  |  |  |  | |  |  |  |  |  |  |  |  |  |  |  | |  | | |
| **Dominant disease site** | Liver | 66% |  |  | | **0.02** | 71% |  |  | 0.24 | 74% |  |  | 0.16 |  |  | | **0.03** | | |
|  | Bone |  |  |  | |  | 100% | NE |  |  |  |  |  |  | NE |  | |  | | |
|  | Primary | 33% | 0.26 | (0.02, 3.18) | |  | 75% | 1.20 | (0.11, 12.9) |  | 75% | 1.04 | (0.10, 11.3) |  | 0.50 | (0.07, 3.74) | |  | | |
|  | Nodal | 100% | NE |  | |  | 100% | NE |  |  | 100% | NE |  |  | 1.33 | (0.18, 9.99) | |  | | |
|  | Disseminated | 20% | 0.13 | (0.02, 0.71) | |  | 47% | 0.35 | (0.10, 1.22) |  | 47% | 0.30 | (0.09, 1.07) |  | 2.78 | (1.34, 5.74) | |  | | |
|  |  |  |  |  | |  |  |  |  |  |  |  |  |  |  |  | |  | | |
| **Number of lesions** | 1 - 4 | 67% |  |  | | 0.92 | 100% |  |  | 0.18 | 100% |  |  | 0.20 |  |  | | 0.12 | | |
|  | 5 - 20 | 56% | 0.63 | (0.05, 7.67) | | (trend 0.75) | 63% | NE |  | (trend 0.51) | 65% | NE |  | (trend 0.45) | NE |  | |  | | |
|  | >20 | 54% | 0.58 | (0.04, 8.15) | |  | 67% | NE |  |  | 67% | NE |  |  | NE |  | |  | | |
|  |  |  |  |  | |  |  |  |  |  |  |  |  |  |  |  | |  | | |
| **Size dominant lesion** | ≤ 5 cm | 70% |  |  | |  | 76% |  |  |  | 79% |  |  |  |  |  | |  | | |
|  | > 5 cm | 35% | 0.23 | (0.07, 0.77) | | **0.01** | 52% | 0.35 | (0.11, 1.09) | 0.07 | 52% | 0.29 | (0.09, 0.95) | **0.04** | 1.90 | (0.95, 3.80) | | 0.07 | | |
|  |  |  |  |  | |  |  |  |  |  |  |  |  |  |  |  | |  | | |
| **Whole body retention** | ≤ 16 | 56% |  |  | |  | 70% |  |  |  | 69% |  |  |  |  |  | |  | | |
|  | > 16 | 56% | 1.00 | (0.33, 3.06) | | 1.00 | 62% | 0.69 | (0.23, 2.10) | 0.51 | 66% | 0.84 | (0.27, 2.62) | 0.77 | 2.07 | (1.02, 4.21) | | **0.04** | | |
|  |  |  |  |  | |  |  |  |  |  |  |  |  |  |  |  | |  | | |
|  | As a continuous variable |  | 0.98 | (0.92, 1.04) | | 0.44 |  | 0.95 | (0.89, 1.01) | 0.09 |  | 0.96 | (0.90, 1.02) | 0.21 | 1.06 | (1.02, 1.10) | | **0.002** | | |
|  |  |  |  |  | |  |  |  |  |  |  |  |  |  |  |  | |  | | |
| **Grade of tumour differentiation** | Grade 1 (Ki67 index < 3%) | 50% |  |  | |  | 71% |  |  |  | 71% |  |  |  |  |  | |  | | |
|  | Grade 2 (Ki67 index 3% - 20%) | 59% | 1.44 | (0.24, 8.84) | | 0.69 | 64% | 0.71 | (0.11, 4.44) | 0.71 | 67% | 0.80 | (0.13, 5.07) | 0.81 | 1.19 | (0.40, 3.55) | | 0.76 | | |
|  |  |  |  |  | |  |  |  |  |  |  |  |  |  |  |  | |  | | |
| **Planned LuTate cycles** | 3 | 39% |  |  | |  | 61% |  |  |  | 59% |  |  |  |  |  | |  | | |
|  | 4+ | 66% | 3.00 | (0.91, 9.92) | | 0.07 | 71% | 1.54 | (0.51, 4.71) | 0.45 | 74% | 1.92 | (0.61, 6.02) | 0.26 | 0.76 | (0.37, 1.54) | | 0.44 | | |
|  |  |  |  |  | |  |  |  |  |  |  |  |  |  |  |  | |  | | |
| **Prior InTate treatment** | 0 cycles | 57% |  |  | |  | 66% |  |  |  | 69% |  |  |  |  |  | |  | | |
|  | 1+ cycle | 55% | 0.93 | (0.30, 2.92) | | 0.91 | 68% | 1.11 | (0.37, 3.39) | 0.85 | 67% | 0.91 | (0.29, 2.82) | 0.87 | 1.17 | (0.58, 2.36) | | 0.66 | | |
|  |  |  |  |  | |  |  |  |  |  |  |  |  |  |  |  | |  | | |
| **Total cumulative LuTate activity (GBq)** | < 31 | 52% |  |  | |  | 63% |  |  |  | 62% |  |  |  |  |  | |  | | |
|  | ≥ 31 | 60% | 1.38 | (0.45, 4.25) | | 0.57 | 70% | 1.38 | (0.45, 4.18) | 0.57 | 74% | 1.75 | (0.56, 5.47) | 0.33 | 0.91 | (0.45, 1.81) | | 0.78 | | |
|  |  |  |  |  | |  |  |  |  |  |  |  |  |  |  |  | |  | | |
| **Concurrent 5FU chemotherapy** | 0 cycles | 40% |  |  | |  | 0% |  |  |  | 0% |  |  |  | 0.40 | (0.14, 1.15) | | **0.08** | | |
|  | 1+ cycle | 58% | 2.05 | (0.31, 13.5) | | 0.45 | 72% | NE |  | **0.002** | 72% | NE |  | **0.007** |  |  | |  | | |
|  |  |  |  |  | |  |  |  |  |  |  |  |  |  |  |  | |  | | |
| **Treatment duration (weeks)** | ≤ 21 | 55% |  |  | |  | 63% |  |  |  | 65% |  |  |  |  |  | |  | | |
|  | > 21 | 58% | 1.13 | (0.36, 3.59) | | 0.83 | 73% | 1.58 | (0.49, 5.04) | 0.44 | 73% | 1.45 | (0.45, 4.70) | 0.53 | 0.81 | (0.40, 1.64) | | 0.55 | | |
|  |  |  |  |  | |  |  |  |  |  |  |  |  |  |  |  | |  | | |
| **Biochemical response** | NA |  | NA |  | |  |  | NA |  |  | NA |  |  |  | 0.20 | (0.09 0.45) | | **<0.001** | | |
| **Molecular imaging response** | NA |  | NA |  | |  |  | NA |  |  | NA |  |  |  | 0.08 | (0.04, 0.19) | | **<0.001** | | |
| **CT response** | NA |  | NA |  | |  |  | NA |  |  | NA |  |  |  | 0.06 | (0.03, 0.14) | | **<0.001** | | |

NA= not applicable; NE= not estimable (HRs were not estimable when no deaths were observed in one of the groups being compared; ORs were not estimable when the response rate was 0% or 100% in one of the groups being compared)

**Supplementary table. Factors associated with objective response (biochemical, molecular imaging, CT) at 6-12 months post induction treatment for patients with previously progressive disease (n=58) and overall survival (n=68)**
